# Recent advances in the application of 2-dimensional gas chromatography with soft and hard ionisation time-of-flight mass spectrometry in environmental analysis

**DOI:** 10.1039/c6sc00465b

**Published:** 2016-04-28

**Authors:** Mohammed S. Alam, Roy M. Harrison

**Affiliations:** a School of Geography, Earth and Environmental Sciences, University of Birmingham Edgbaston Birmingham B15 2TT UK r.m.harrison@bham.ac.uk

## Abstract

Two-dimensional gas chromatography has huge power for separating complex mixtures. The principles of the technique are outlined together with an overview of detection methods applicable to GC × GC column effluent with a focus on selectivity. Applications of GC × GC techniques in the analysis of petroleum-related and airborne particulate matter samples are reviewed. Mass spectrometric detection can be used alongside spectral libraries to identify eluted compounds, but in complex petroleum-related and atmospheric samples, when used conventionally at high ionisation energies, may not allow differentiation of structural isomers. Available low energy ionisation methods are reviewed and an example given of the additional structural information which can be extracted by measuring mass spectra at both low and high ionisation energies, hence greatly enhancing the selectivity of the technique.

## Abstract 2

Two-dimensional gas chromatography has huge power for separating complex mixtures.

## Graphical abstract

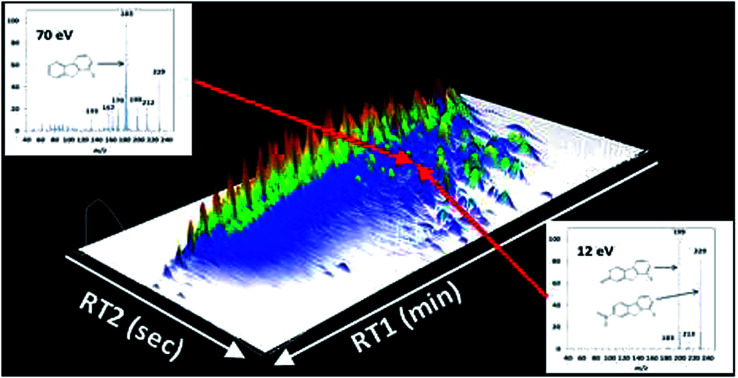

## Introduction

Airborne particulate matter is a subject of intense current research driven largely by its adverse impacts upon human health^1–3^ and its importance in global climate regulation.^4,5^ Road traffic makes a substantial contribution to airborne particulate matter through direct emission of particles (especially from diesels) and through emissions of gases such as oxides of nitrogen which are oxidized to form particles.^6,7^ A further mechanism which has recently been recognised as making a very significant contribution to airborne particulate matter is the emission of particles containing a substantial semi-volatile organic component which vaporises as the particles move downwind from the source and which are then oxidised, both contributing to formation of ground level ozone and forming a substantially larger mass of particles of more highly oxidized compounds, referred to as secondary organic aerosol.^8^ Current atmospheric chemistry-transport models focus very heavily on the volatile organic compounds (generally C_10_ or less) while ignoring the higher molecular weight compounds for which very little information currently exists. This provides a severe limitation on the capability of such models to provide reliable predictions of formation of both ozone and secondary organic aerosol.

The primary reason why very little work has been conducted on hydrocarbons of greater than C_10_ is that as a consequence of their huge diversity conventional gas chromatography is unable to provide a separation and these compounds appear in the chromatogram as a large hump referred to as unresolved complex mixture (UCM). The advent of two dimensional gas chromatography (GC × GC) techniques in recent years has provided a means to disaggregate the UCM hump providing separate elution of literally thousands of compounds which can be characterised on the basis of their mass spectra. This mini review article explores the use of GC × GC in airborne and petroleum samples and discusses recent advances in soft and hard ionisation time of flight mass spectrometry.

### Overview of principles

Since GC × GC was first introduced more than 20 years ago, it has become the powerful analytical technique of choice when resolving complex mixtures. A number of reviews have been published over the last two decades, initially focusing on the principle and experimental technique^9–13^ followed by its application.^14–18^ GC × GC is a hyphenated chromatographic technique involving the coupling of two columns connected sequentially, with a modulator positioned between them. The modulator, located at the head of the second column, transfers fractions of the effluent from the primary column (generally non-polar) to the secondary column (generally polar), providing an enhanced peak capacity and separation power owing to the orthogonal separation by the two differing properties of the columns. The modulator must detain a fraction of the effluent, refocus and rapidly release it onto the second column in a narrow band ensuring maximum resolution. There have been a number of papers describing different modulation techniques including thermal modulators with heater based interfaces,^19,20^ which trap primary column effluent at or above ambient temperatures; thermal modulators with cryogenic based interfaces,^21–23^ trapping primary column effluent below ambient temperatures; valve based modulators^24,25^ which exploit pneumatic valve systems to achieve modulation of the primary column effluent; and more general review articles.^14,17,18,26^

### Separation of compound classes

Compounds belonging to the same chemical group in a mixture possess similar physicochemical properties. This facilitates identification when separated according to these physical and chemical properties as this provides structured distribution patterns of chemical groups in an ordered appearance in the chromatogram. This is an advantage of the GC × GC technique. The use of two dimensional retention data for group separations is well established.^27,28^ However, to positively identify peaks within a chromatogram solely relying upon group type pattern separation, the use of pure standard compounds and retention indices is inconceivable. A greater amount of information is obtained from GC × GC when coupled to a suitable detector.

Various detection methods for GC × GC are described hereafter. Briefly, the preferred method of detection for GC × GC in recent years is time-of-flight mass spectrometry (TOFMS), where each chromatographically resolved peak possesses a unique full mass spectrum. Traditional mass spectrometers (MS) employ electron impact (EI) ionisation at 70 eV. The electron imparts a large amount of excess energy when ionising a molecule, resulting in extensive fragmentation. The fragmentation patterns are identified by comparing them to mass spectral libraries. However, due to the lack of the molecular ion signal and non-specific fragmentation patterns of organic species (*e.g.* aliphatic hydrocarbons), many compounds remain indistinguishable. Thus there is a continual demand for more robust soft (low energy) ionisation techniques for MS, in order to retain the molecular ion signal and aid identification of spectra. This study reviews the applications of GC × GC techniques in the analysis of petroleum-related and airborne particulate matter samples with a focus on recent developments in soft ionisation TOFMS.

## Detection methods for GC × GC

Due to the second dimension separation being inherently narrow, the detector must be capable of acquiring data with a high sampling rate; with the optimum being in the 50–100 Hz range. There are many commercially available detectors for GC × GC, some of them which are reviewed below.

### Flame ionisation detection (FID)

In the preliminary years of GC × GC, FID was the preferred method of detection, as applications were in the field of petrochemical analysis.^29,30^ The FID technique has negligible internal volumes and has been demonstrated to collect data at frequencies of up to 300 Hz.^31^ Although FID is stable over long periods of time and is easily calibrated, it reveals no structural information on compounds of interest.^17^ FID gives a general response for hydrocarbons while displaying no response for major atmospheric gases allowing it to be deployed in field measurements.^32,33^

### Electron capture detection (ECD)

ECD is a highly selective and sensitive technique detecting electron absorbing components. The suitability of ECD detection for GC × GC was first investigated in the late 1990s where one important aspect under investigation was the contribution of the cell volume of these detectors to the band broadening of the eluting peaks.^34^ Kristenson *et al.*^35^ compared commercially available ECDs and found that only the micro-ECD (internal volume of 150 μL) possessed reasonable results. However, for satisfactory performances high detector temperatures in the range of 320–350 °C and high gas flows (150–450 mL min^−1^) are desirable. Due to the ECD's limited dynamic range, its greatest application is in the analysis of halogen containing compounds, including polychlorinated biphenyls (PCBs),^36–38^ pesticides,^39^ and chlorinated paraffins.^40^

### Sulphur chemiluminescence detection (SCD)

The SCD was first developed by Benner and Stedman^41^ and was applied to GC by Shearer *et al.*^42^ It exploits the chemiluminescent reaction of SO + O_3_ and does not suffer from quenching and interferences and has a universal response to all organosulphur compounds over a wide dynamic range.^42,43^ It became the detector of choice for detailed analysis of organosulphur compounds after the introduction of the flameless burner.^43^ SCD was coupled to GC × GC after the availability of the commercial flameless burner which can be used at 800 °C, with collection of the emitted light (260–480 nm) at a sampling rate of 50 Hz. This technique has since been used to analyse sulphur containing compounds in diesel,^44^ middle distillates,^45^ petroleum source rocks^46^ and process waters.^47,48^

### Nitrogen chemiluminescence detection (NCD)

NCD utilises a similar principle of operation as SCD (*i.e.* chemiluminescent reaction of NO + O_3_), and generally produces a linear and equimolar response to nitrogen containing compounds. Wang *et al.*^49^ speciated nitrogen compounds in diesel fuel using GC × GC-NCD reporting a sampling rate of 100 Hz for the detector. Adam *et al.*^50^ compared two commercially available NCD instruments with different flameless burners and showed that only one of the NCD instruments demonstrated the required acquisition frequency and thus was suitable for GC × GC. NCD has since been utilised in atmospheric^51^ and food samples.^52,53^

### Nitrogen phosphorus detection (NPD)

NPD (also referred to as specific thermionic ionisation detection) was first investigated as a GC × GC detector by Ryan and Marriott.^54^ It is composed of a bead sensor doped with an alkali metal salt and attached to an electrically heated wire. This serves as the thermionic source where due to surface ionisation effects, alkali metal atoms are ionised by collision with plasma particles. The response of the detector is highly dependent upon the nature of the optimisation of the gas environment immediately surrounding the thermionic surface which can be a major drawback.^54^ When the air is mixed with a low nitrogen flow, the plasma provides specificity for nitrogen and phosphorus containing compounds. NPDs fast data acquisition rate of 100 Hz has enabled it to be used in analysis of atmospheric samples,^55^ heavy gas oil,^56^ fungicide residues in vegetable samples^57^ and incense.^58^

### Mass spectrometry (MS)

#### Quadrupole mass spectrometry (qMS)

MS is most often coupled to GC × GC allowing another dimension to classify compounds. MS ensures high selectivity throughout the chromatogram and provides structural information for unambiguous identification. Several compound classes demonstrate unique fragmentation patterns in the mass spectrum and thus give valuable information about compounds, which can be compared to spectral libraries in the literature (*e.g.* NIST). Many studies have attempted to couple a qMS to GC × GC, with reasonable results.^59,60^ However, the general consensus is that acceptable results can only be obtained for a restricted mass range of up to *ca.* 300 Da and a data acquisition rate of approximately 10–33 Hz (ref. 14 and 15).

#### Time-of-flight mass spectrometry (TOFMS)

Faster data acquisition rates are possible when coupling a GC × GC to TOFMS, where up to 500 spectra per s can be obtained (a single spectrum consists of 10 pulses).^28^ Spectral deconvolution is also possible due to the high speed full spectrum acquisition rates, without mass spectral skewing across the chromatographic peak. There are a number of commercially available GC × GC-TOFMS systems used both in the research and industrial laboratories. The high acquisition rate data files generated by TOFMS systems are large, and automated detection of peaks and data presentation are both complex and time consuming. Search criteria of specific ions and rules for GC × GC-TOFMS have been reported by numerous studies.^61–66^

#### Soft ionisation techniques

Several soft ionisation techniques have been developed with MS, including chemical ionisation (CI),^67,68^ field ionisation (FI),^69,70^ and photoionisation (PI),^71,72^ and have been reviewed recently.^73^ Maccoll and co-workers have reported low EI (12.1 eV) and low temperature (350 K) mass spectra of various compound classes in a number of publications,^74^ since their early work on ion enthalpies and their application in MS.^75^ Very few of these soft ionisation mass spectrometers, however, have been coupled to GC × GC.^76–78^

## Application of GC × GC

### Petroleum products

Crude petroleum and the fractions derived from it in refining have huge chemical complexity, and GC × GC offers many advantages as an analytical tool. There have been a number of overview papers describing the application of GC × GC techniques to petrochemical and related samples, including crude oil in the environment^79,80^ and more general review articles.^14,15^ For the hydrocarbon constituents, the FID is a viable detector for substances which are clearly separated and for which retention times on both columns are well known after calibration with standard compounds. It also finds application when groups of compounds or homologous series are quantified together. However, for more complex mixtures, and when, as is often the case, unknown compounds are present, the mass spectrometer detector offers great advantages. The rapid scan rate of the time-of-flight mass spectrometer is often an asset.

GC × GC-FID has been applied to the analysis of a wide range of compound types in crude oils,^81,82^ as well as many products derived from crude oil, such as jet fuel,^83^ naphtha,^84,85^ diesel,^86^ gasoline,^87,88^ middle distillates^89^ and vacuum gas oil.^90^ In some cases, such as the latter,^90^ rather than identifying individual compounds, the chromatogram is used to identify and quantify compound groups (*e.g.* saturates, monoaromatics, diaromatics, *etc.*) and volatility profiles within those groups.

The mass spectrometer detector, usually a TOFMS, but in some instances a quadrupole, adds considerable capability, and this has been applied to chemical compounds within crude oils,^28^ gasoline,^87^ marine diesel fuel,^91^ aromatic compounds in extra heavy gas oil,^92^ aromatic steroids and hopanoids in crude oils,^93^ biomarkers (hopanes, steranes and terpanes) in crude oils,^94^ and complex hydrocarbon mixtures in steam cracking plant effluent to which FID detection was also applied.^95^ Von Mühlen *et al.*^96^ used GC × GC-TOFMS to identify firmly 120 N-containing compounds, and tentatively a further 108 such compounds in heavy gas oil petroleum fractions. GC × GC-FID and GC × GC-TOFMS have also been applied to other fuel types, including oils derived from pyrolysis of biomass, containing mainly polar oxygenated compounds,^97^ fatty acid methyl esters in biodiesel/petroleum diesel blends,^98^ sulphur compounds in coal tar,^99^ and hydrocarbons in coal liquids.^100^

Environmental degradation adds to the complexity of oil-derived material, and GC × GC methods have found applications in the analysis of processed crude oil^101^ and biodegradation products of petroleum in the environment.^102–104^ Contaminated soils and leaching water analyses have been reported. Van de Weghe *et al.*^60^ report the application of GC × GC-FID to oil-contaminated soils, and Mao *et al.*^105^ used GC × GC-FID analyses in conjunction with ecotoxicity tests in soils, and in ecotoxicity assays of petroleum hydrocarbon degradation and soil and leaching water.^106^ Seeley *et al.*^107^ used a GC × GC-FID system capable of operation at up to 340 °C for the analysis of diesel fuel, gas oil, motor oil and extracts of petroleum contaminated water, wastewater and soil samples. GC × GC methods were also used to resolve thousands of compounds in the UCM derived from extraction of petroleum-contaminated sediments,^108^ and in biopiles used to remediate petroleum-contaminated soils.^109^

Some workers have used chromatographic separations prior to GC × GC analysis to reduce the complexity of samples. Hence Mao *et al.*^105,106^ used a silver-modified HPLC column prior to GC × GC which allowed separation of petroleum hydrocarbons in the middle distillate range (C_8_–C_40_) into nine groups: alkanes, alkenes, cycloalkanes, monoaromatics, naphthenic aromatics, diaromatics, naphthenic diaromatics, triaromatics and >3 ring polycyclic aromatics, which were quantified with a FID after GC × GC separation. Vendeuvre *et al.*^85^ employed an olefin trap upstream of a GC × GC-FID to allow a cleaner separation of saturates and olefins in a heavy naphtha (C_8_–C_14_ range), and Edam *et al.*^86^ achieved a cleaner separation of aromatic and naphthenic compounds by using a prior liquid chromatography separation. Van Stee *et al.*^110^ gained selectivity in GC × GC analysis by use of an atomic emission detector. In application to petrochemical analysis, they recorded traces for C, H, Cl, Br, Si, N and P. In a case study, a wide range of S-containing compounds were analysed in a crude oil and a fluidised catalytic cracking product.

Crude oils from different reservoirs within one oil field typically show only minor differences in composition, and van Mispelaar *et al.*^111^ report the application of multivariate statistical techniques to discrimination between highly similar samples. Some peaks were found to vary between samples, while the majority did not, and 292 peaks were used in developing a discrimination model.

### Airborne particulate matter

Petroleum fuels and oils are a major source of hydrocarbons in the atmosphere, both from fuel evaporation and from engine exhaust emissions. Compounds range in volatility from highly volatile low molecular weight hydrocarbons classified as VOC, through intermediate and semi-volatile compounds which actively partition between the condensed and vapour phases, to low volatility compounds which associate almost entirely with airborne particles. All such compounds are liable to atmospheric chemical processing which leads to increased O : C and N : C ratios and further adds to the complexity of the mixture.

Arsene *et al.*^18^ have reviewed the application of hyphenated GC × GC-MS techniques for the analysis of volatile organic compounds in air. After an initial focus on instrumental considerations, they review published studies, including both vapour and particulate phases. Hamilton^17^ in another review article describes the instrumental hardware, and its application to gas phase species, aerosols and simulation chamber experiments. Calibration and data analysis methods are also reviewed.^17^ One of the earliest applications of the GC × GC method to atmospheric samples was the analysis of more than 500 volatile organic species by Lewis *et al.*^112^ Xu *et al.*^33^ optimised a GC × GC-FID system to resolve C_7_–C_14_ organic components, and also applied a TOFMS detector for compound identification. A similar system was deployed in Crete for measurement of C_7_–C_11_ aromatic and *n*-alkane hydrocarbons,^113^ and on Tenerife for analysis of terpenes.^114^ Bartenbach *et al.*^115^ used GC × GC-FID to analyse hydrocarbons from C_6_ to C_8_, α and β-pinene, 3-carene, camphene and eucalyptol in the atmosphere, showing a generally fair to good correlation (*r*^2^ = 0.41–0.88) with GC-MS. Hamilton and Lewis^116^ applied both FID and TOFMS detection in the analysis of monoaromatic compounds in gasoline and urban air. They report finding 147 monoaromatic species in urban air, with up to eight carbon substituents on the ring. A number of oxygenated species were also reported.^116^ Dunmore *et al.*^117^ applied a GC × GC-FID to measurement of hydrocarbons in the air of London. Compounds from C_6_ to C_13_ were determined and found to make an appreciable contribution to the VOC content of urban air, and its ability to react with hydroxyl radical. This was considered to be an important source of secondary organic aerosol, and many oxidised compounds were also detected.^117^ Goldstein *et al.*^118^ describe the development of a new instrument, 2D-TAG, in which an *in situ* thermal desorption aerosol instrument is interfaced with GC × GC. The instrument is able to make automated hourly measurements of atmospheric particulate matter using an FID or quadrupole MS as detector. Worton *et al.*^119^ describe the coupling of the thermal desorption aerosol gas (TAG) system with GC × GC-TOFMS analysis to give hourly measurements of speciated organic compounds in atmospheric aerosols. Various instrumental enhancements were also described.

Most studies have used a TOFMS detector, and for example, Hamilton *et al.*^62^ identified around 130 specific oxidised VOC compounds, and more than 100 further such compounds lacking positive identification in samples of urban airborne particles. The same group used direct thermal desorption of airborne particulate matter to identify between 17 and 57 organonitrogen compounds in 23 urban air samples, with in total 100 different organonitrogen compounds identified ranging in molecular weight from 59–302 Da and containing from 1–4 nitrogen atoms.^63^ A large suite of polycyclic aromatic compounds (hydrocarbons, oxygenates and nitro-compounds) were identified in standard reference material by GC × GC-MS/MS, and quantified successfully in particles sampled from diesel exhaust.^120^ Polycyclic aromatic hydrocarbons and oxygenated polycyclic aromatic hydrocarbons were also analysed in urban particulate matter samples by GC × GC-FID and GC × GC-QMS.^59^ Mass spectrometer detection was recommended for identification, while the FID was used for quantification. Amador-Muñoz *et al.*^121^ used an isotope dilution method with GC × GC-TOFMS to characterise polycyclic aromatic hydrocarbons in urban dust reference material (SRM 1649a). Kallio *et al.*^122^ applied GC × GC-TOFMS to the identification of organic compounds in atmospheric aerosols from a coniferous forest. Mass spectra and retention indices were used to identify around 50 compounds, including acyclic alkanes, alkenes, ketones, aldehydes, alcohols, acids, aromatics and oxidised monoterpenes. Biomass burning is another large source of organic compound emissions and Hatch *et al.*^123^ used GC × GC-TOFMS to measure 708 positively or tentatively identified compounds in biomass smoke. Both the vapour and particle phases were analysed, and emission factors calculated.

Ochiai *et al.*^55^ used both a quadrupole mass spectrometer (QMS) detector, a nitrogen–phosphorus detector, and a high resolution (0.05 Da) time-of-flight detector to analyse nanoparticles from roadside air after thermal desorption. A wide range of compounds were identified with the HR-TOFMS. The QMS showed good linearity and repeatability and allowed quantitative analysis of polycyclic aromatic hydrocarbons. GC × GC-TOFMS was used by Alam *et al.*^64^ for the analysis of urban particulate matter after solvent extraction with dichloromethane/methanol. Many different compounds were identified from the spectral library, with an emphasis upon complex industrial chemicals in the C_6_–C_21_ range, many of which were oxygenated, with some also containing nitrogen.

Welthagen *et al.*^61^ describe the application of direct thermal desorption and GC × GC-TOFMS to the characterisation of semi-volatile organic compounds in the PM_2.5_ size fraction of particulate matter sampled in Augsburg, Germany, with more than 15 000 peaks detected. The same team report the daily quantification and semi-quantification of 200 compounds including *n*-alkanes, *n*-alkan-2-ones, *n*-alkanoic acid methyl esters, acetic acid esters, *n*-alkanoic acid amides, nitriles, linear alkylbenzenes and 2-alkyltoluenes, hopanes, PAH, alkylated PAH and oxidised PAH as well as some compounds not belonging to these compound classes.^124^ In a subsequent paper, Vogt *et al.*^125^ recommend a partial classification system which uses fragmentation patterns, retention times and spectral transformations for automated classification.

GC × GC methods have also been applied in the analysis of reaction products in chamber experiments. Hamilton *et al.*^116^ analysed photo-oxidation products from a series of alkylbenzenes in cryo-focused air samples withdrawn from the European Photoreactor chamber (EUPHORE). A wide range of oxygenates was found. In a similar set of experiments, Webb *et al.*^126^ studied the oxidation of *o*-tolualdehyde, finding a range of products, including oxygenated polycyclic aromatic hydrocarbons. Products from the smog chamber oxidation of the sesquiterpene longifolene were characterised by Isaacman *et al.*^127^ using a GC × GC Aerosol Gas Chromatograph/Mass Spectrometer (2D-TAG) instrument. Nearly 200 oxidation products were observed, many of which could not be characterised due to a lack of standards, and their absence from mass spectral databases. The product distribution was seen to evolve with time due to continuing oxidation processes.

## Recent advances in application of GC × GC analysis

### Soft ionisation as a separation tool

The limitations of EI have led to a demand for soft ionisation techniques for MS. Korytár *et al.*^128^ exploited GC × GC coupled to electron capture negative ionisation TOFMS (ECNI-TOFMS) to analyse C_8_–C_14_ polychlorinated *n*-alkane (PCA) congeners in dust. The authors report the potential inability of GC × GC alone to identify a large number of PCA congeners that display coelution, and demonstrate that ECNI can be used to identify diastereoisomers. Wang *et al.*^129^ reported a two dimensional separation approach of diesel fuel exploiting GC-FI-MS and compared it to that of GC × GC. The authors were able to use soft ionisation MS to achieve good compound class separation on the basis of their parent masses. Hejazi *et al.*^130^ developed a method that utilises GC with parallel EI and FI-MS that consists of two mass spectrometers connected to a single GC. The dual source instrument generates equivalent chromatograms aligned in time, allowing accurate assignment of fragment ions (from EI) to the corresponding molecular ions (from FI).

Zimmermann and co-workers^131^ have demonstrated the applicability of two fragmentation free PI techniques; resonance-enhanced multi-photon-ionisation (REMPI) and single photon ionisation (SPI) with MS. REMPI uses intense UV light laser pulses for a two photon ionisation process, and is highly sensitive and selective to aromatic and polyaromatic compounds. It is therefore not applicable to use REMPI for the analysis of petroleum samples that are rich in aliphatic compounds. SPI on the other hand utilises VUV photons for the ionisation and is capable of ionising all organic compound classes including compounds found in petroleum samples. Ferge *et al.*^132^ reported fragmentation free ionisation of long chain alkane, alkanoic acid, aromatic hydrocarbon, oxygenated-PAH and nitroaromatic compounds using LD-SPI-TOFMS from spiked particulate matter filter samples. Streibel *et al.*^133^ applied thermal desorption (TD) at 120, 250 and 340 °C, for consistency with OC/EC measurement methods, to assess the organic content of urban aerosol. After desorption both REMPI and SPI methods were coupled to TOFMS. The authors claim that allotment of organic species on a molecular level to fractions of organic carbon is possible with this method. Recently, the chemical composition and aromatic emission profiles were studied using a TOC analyser coupled to REMPI-MS.^134^ PAH, oxy-PAH and alkylated-PAH were identified in the emissions which reflected the types of fuels used (heavy fuel oil and distillate fuel). Welthagen *et al.*^76^ demonstrated a three dimensional separation technique by coupling GC × GC with SPI-TOFMS to analyse petroleum diesel samples. The authors found that detector was unable to achieve a fast data acquisition rate due to the repetition rate of the pulsed laser only being 10 Hz. However, using an electron beam pumped excimer lamp (EBEL) as the VUV light source the SPI-TOFMS as a detector was significantly enhanced. The use of soft ionisation by laser photo-ionisation is shown to enhance the selectivity of GC × GC.^76^

Goldstein and co-workers have also demonstrated the applicability of VUV SPI-MS in a number of studies. For example, Chan and co-workers^78^ applied GC × GC with VUV photoionisation and mass spectrometric detection to the analysis of the unresolved complex mixture of organic compounds in the atmosphere. They reported it to be the most detailed characterisation of UCM composition in atmospheric samples to date. The low energy of the VUV system in comparison to traditional 70 eV electron ionisation gave less fragmentation of the molecular ions and combined with retention times proved valuable in distinguishing *n*-alkanes, branched alkanes, bicycloalkanes, tricycloalkanes, steranes, hopanes, benzenes and tetralins of the same carbon number. Composition data were used to infer sources of hydrocarbons, and to estimate rate coefficients for OH radical attack on branched alkanes by measuring *n*-alkane : branched alkane ratios during transport of polluted air masses.^78^ Isaacman *et al.*^135^ reported the improved resolution of hydrocarbon structures and constitutional isomers in diesel fuel using GC-VUV-MS. The composition of a diesel fuel sample as a fraction of the total observed mass of each double bond equivalent (DBE) class at each carbon number in the range C_15_–C_25_ was 73% aliphatic and 27% aromatic compounds. A wide range of compounds were positively identified including, saturated and unsaturated aliphatic hydrocarbons, hopanes, steranes, PAH, aliphatic ketones and aldehydes, oxygenated and multifunctional aromatics and acids and esters. The role of lubricating oil in primary organic aerosol emissions^136^ and heterogeneous OH oxidation of lubricating oil^137^ has also been investigated using VUV PI from the same laboratory. More recently, C_9_–C_33_ hydrocarbons were comprehensively characterised from NIST SRM 2779 Gulf of Mexico crude oil with a mass balance of 68 ± 22% using GC/VUV-MS.^138^ The authors highlight the technique in overcoming the necessity for individual compounds to be chromatographically resolved in order to be characterised. Drawbacks of SPI VUV, however, are the relative experimental complexity of the technique and the reduced stability of the molecular ions that are formed (radical cations) in comparison to other techniques such as MALDI and atmospheric pressure CI (even-electron ions).^72^

Recently, the commercially available BenchTOF-Select (Markes International, Llantrisant, UK) has been introduced into the market, demonstrating variable ionisation energies from 10–70 eV. Each sample can be analysed using different ionisation energies by means of repeat injections. Conventional EI ion sources use a potential difference of 70 eV to accelerate electrons from the surface of a negatively charged filament to a positively charged ion chamber. The BenchTOF-Select uses ion optics to retain this high potential difference, but reduces the accelerating electrons energy prior to arriving at the ion chamber. This allows the ionisation energy to be varied in the range 10–70 eV. Unlike CI and FI, no reagent gases, adjustments in pressure or switching between sources are required. We evaluate this detector in the following section analysing urban aerosol samples by coupling the detector to comprehensive two dimensional GC (7890B, Agilent Technologies, Wilmington, DE, USA) equipped with a Zoex ZX2 modulator (Houston, TX, USA). The first and second dimension were equipped with a SGE DBX5 non-polar capillary column (30 m, 0.25 mm ID, 0.25 μm – 5% phenyl polysilphenylene-siloxane), and a SGE DBX50 (4.0 m, 0.1 mm ID, 0.1 μm – 50% phenyl polysilphenylene-siloxane) column, respectively. The GC × GC was interfaced with a BenchTOF-Select, time-of-flight mass spectrometer (TOFMS, Markes International, Llantrisant, UK). The scan speed was 50 Hz with a mass resolution of >1200 FWHM at 70 eV and >800 FWHM at 14 eV over 100–1000 *m*/*z*. The mass range was 35 to 600 *m*/*z*. 24 hour aerosol samples were collected using a high volume Digitel Sampler at a roadside site in Birmingham. As the purpose of this study is to evaluate this detector, further information in regards to sample preparation, collection and extraction can be found elsewhere^64^ as well as information regarding the site location.^139^

**Fig. 1:**
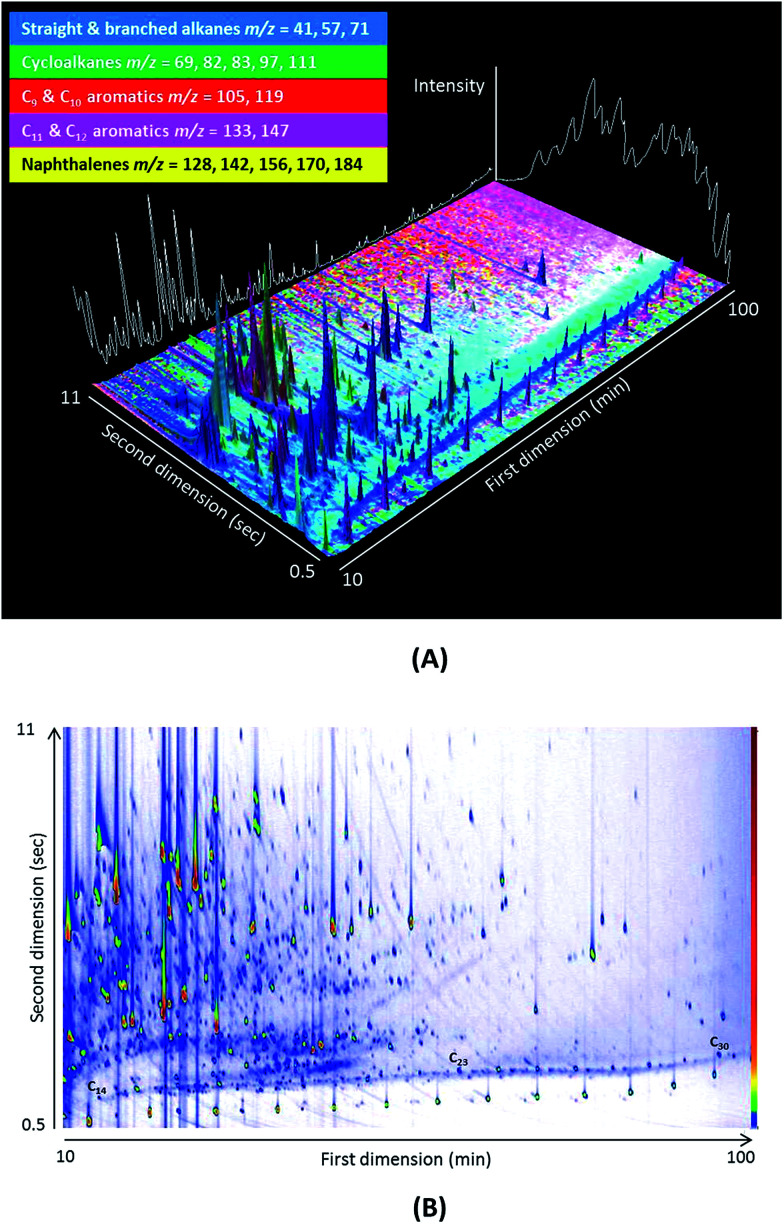
(A) A 3D representation of a GC × GC chromatogram of an urban aerosol filter sample. The different colours represent different *m*/*z* ratios. (B) Contour plot produced by GC Image v2.4.

A typical two-dimensional separation at 12 eV is presented in Fig. 1(A) and (B) for an aerosol sample. The white line in Fig. 1(A) illustrates the separation achievable using traditional GCMS, while the different colours signify specific *m*/*z* ratios of species. The nature of the ordered chromatograms produced by GC × GC (see *n*-alkane series in Fig. 1(B)) allows compounds to be identified by retention time, while running the TOFMS at 70 eV enables the identification of compounds using mass spectral libraries (which are published at 70 eV). Interpretation is therefore required for mass spectra that are collected at lower ionisation energies, as there is no library for soft ionisation fragmentation, which is a drawback of this methodology.

### Differentiation of structural isomers

**Fig. 2:**
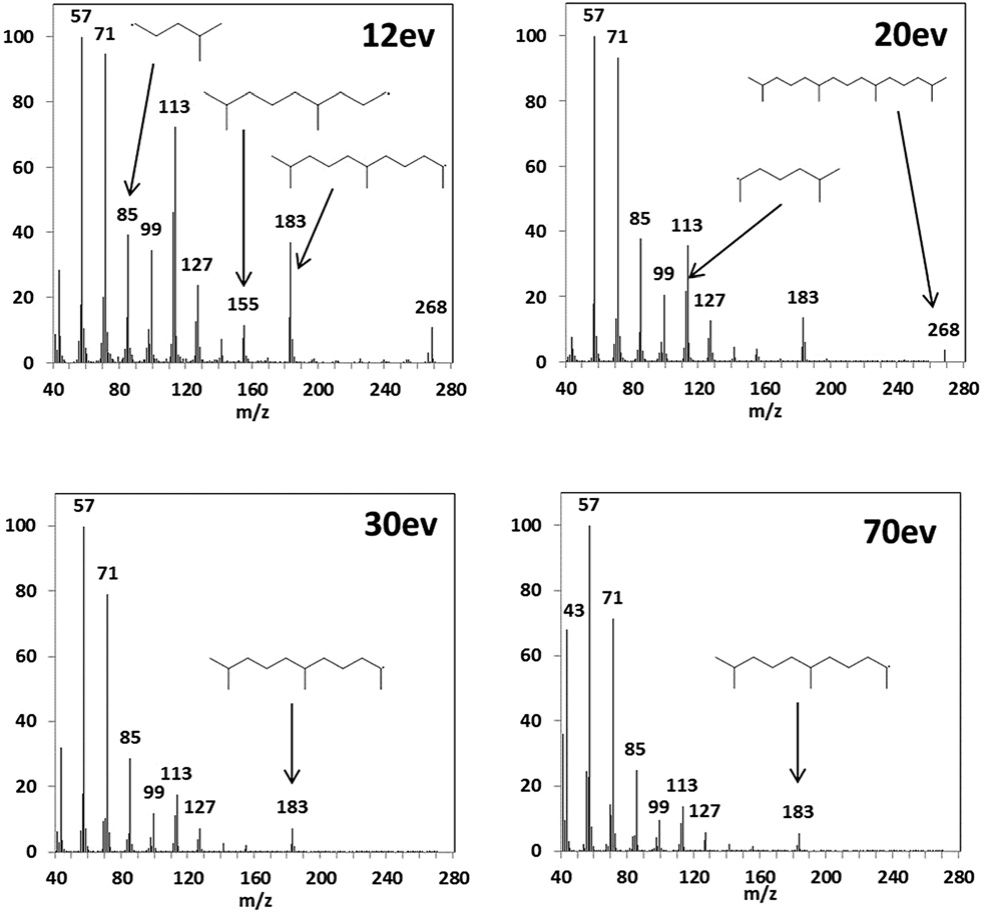
Fragmentation mass spectra of pentadecane, 2,6,10,14-tetramethyl- (*m*/*z* = 268) at 4 different ionisation energies.

Although there are various compound classes that are identified using 70 eV mass spectra including, *n*-alkanes, alkanoic acids, aldehydes and ketones, esters, PAH, oxygenated-PAH, alkylated-PAH, cyclohexanes, steranes and hopanes; structural isomers within each compound class (particularly for larger compounds for which there are an enormous number of possible species^140^) are indistinguishable due to the absence of their molecular ion. Reducing the ionisation energy and obtaining the molecular ion can aid identification of these compounds. To illustrate the difference in fragmentation patterns achievable, the known compound pentadecane, 2,6,10,14-tetramethyl-, which was present in all aerosol samples analysed, was examined using ionisation energies of 12, 20, 30 and 70 eV, shown in Fig. 2. This specific compound was investigated as its mass spectrum is well known, but when present within a complex mixture cannot be distinguished from other branched alkanes (without retention time information), see Fig. 2. The high ionisation energy mass spectra do not show the presence of the molecular ion (*m*/*z* = 268), whereas for 20 and 12 eV spectra both the molecular ion and most prominent fragments are represented by peaks. There was very little difference in the sensitivity when re-analysing the aerosol samples at different ionisation energies.

Furthermore, the separation and identification of structural isomers such as the monomethylalkanes in a broad range of carbon atoms is difficult. This is not only because there are a huge number of isomeric possibilities but also due to coelution when using GC or GC × GC. GC × GC-FID and GC/MS/MS with a 100 m column was utilised to separate 63 C_9_–C_19_ monomethylalkanes in exhaled breath.^141^ Moreover, 196 C_4_–C_30_ monomethylalkanes were identified using GC/MS equipped with a 100 m column using linear retention indices in diesel fuel.^142^ These studies considered a targeted approach in their analyses and completed complex sample preparation, together with long GC runtimes. The use of variable ionisation energy coupled to mass spectrometry, as well as retention time information enables a non-targeted approach to be conducted. Alam *et al.*^143^ recently reported nine C_21_ isomeric monomethyl alkanes by interpreting their respective mass spectra at 14 eV using BenchTOF-Select. The authors demonstrated the applicability of soft ionisation to positively identify the positioning of branching for various aliphatic, monocyclic, bicyclic and tricyclic alkanes.

## Conclusions

There are many applications of GC × GC methods in the analysis of petroleum-related and environmental samples where the technique offers huge improvements in separation capability relative to one-dimensional chromatography. Greatly enhanced selectivity can be achieved through the use of element-specific detectors, but for analysis of hydrocarbons, the options are more limited. The combination of time-of-flight mass spectrometry with variable energy ionisation has the advantage of allowing the identification both of molecular ions and major fragments, hence greatly enhancing the power to identify specific structural isomers.
